# National Guard Or?

**Published:** 1917-03

**Authors:** 


### National Guard Or?

In our issue of Feb. 1916, we expressed the opinion that the National Guard system should be adhered to, instead of creating a new Continental Army. This opinion was not so much a personal one as a compilation of the best that could be obtained from those presumably familiar with conditions. We are not yet disposed to acknowledge that this opinion was an error. It is true that a considerable percentage of the Guard was found not to be up to the physical standards for actual service but no harm was done by accepting these men, either to the Guard for its established functions nor to the men themselves. Those rejected might still be of value if the need of national defense were emergent. It is doubtful if their places would have been filled by others if they had been rejected by the Guard surgeons. The Guard soldiers were, in many states, neither well drilled nor thoroughly equipped nor were the ranks full. But they were a good deal better than nothing and nothing else was available except entirely raw recruits. The Guard was not promptly mobilized at the border nor always properly transported according to peace standards but this was a defect of the national authorities, not of the Guard itself. It has been said that, owing to lack of ammunition, patrols often served with empty guns—but this can scarcely be charged against the Guard. It is said that the Guard has developed a ''never again'' spirit. Political considerations aside, it must be recognized that the patriotism that will enlist only in the presence of immediate danger and that will sacrifice business interests only when immediate fighting is to be indulged in, is of very little protective value. The border service has been of the kind necessary to insure actual training for war and, as we understand Preparedness, it means not so much getting an army together to fight as to have it ready to prevent fighting. The waiting policy at the border has been exactly the kind of training proposed for the Continental Army, plus environment and expectations increasing its realism. On the average, it will even have equaled in length of actual training about what was proposed for the Continental Army so that the ''never again'' spirit, if universal among the returned soldiers will mean only that they will ask discharge as was proposed for the Continental Army, from active service or immediate connection with a military organization.

We do not by any means subscribe to the theory that the border service should have been performed entirely by the regular army, that all such forms of national police work should be thus performed. However large the regular army, however adequate to any such task, similar services should be participated in by the organized militia as a preparation for defense on a large scale if it ever becomes imperative.

The advantages of local organization, with personal acquaintance between officers and men, opportunities for training without interruption of civil pursuits, for furnishing social and athletic incentives for enlistment, for allowing rapid and convenient means of mobilization, for the development of local esprit de corps as well as national patriotism, are all points of practical superiority. The local basis of organization of any protective military force should, if anything, be further emphasized. Every unit should feel secure that it will be mobilized under its own officers and recruited from its own locality, unless there are grave military reasons to the contrary, as during the actual progress of a war of considerable duration. Disregard of this principle has been one of the greatest handicaps of the Guard and will undoubtedly deter enlistment and serious study by officers, after the return to the normal peace conditions existing a year ago.

But, we must face the lessons of practical experience. The Guard can no longer be satisfactorily maintained as a protection against strikes. That form of police duty can be best performed by a definite constabulary. Labor will not, whatever the ethics of the case, consent to oppose its own kind and if the Guard could be sufficiently maintained without the participation of laboring men in the usual sense of the word, there would arise not only an undesirable but a dangerous class antagonism. Both individual and public sentiment demands that the soldier shall not unduly sacrifice his earning capacity, opportunities for promotion, and his family. Either his employers, or the community, unofficially or through taxation, must make up to him and to his family a considerable portion of his loss. This means that a man of fair earning capacity, well started in life and with a family, costs more than he is worth as a private soldier and that it is contrary to public policy to enlist him unless in great emergency. There is also a growing sentiment, in and out of the National Guard that it is not true patriotism to continue a purely voluntary service which allows a general shirking of duty and places on the volunteer not only his own share of patriotism and self-sacrificing, but that of others. Something like universal military service is very widely advocated, not only as a means of national defense on an adequate scale but for the benefits accruing to the community and to the individual in peace. More recently it has been proposed, in N. Y. at least, to do away altogether with state provision for military organization. This would be a radical step but it places the responsibility squarely on the national government, implicitly demands that the burden be equally distributed over the whole country, and that the national military authorities maintain a state of preparedness such as they must even to take over the organized militia in time of need. We also regard it favorably as one step toward the elimination of state governments altogether—which is not entirely an indication of personal folly, since it was contemplated and openly discussed by the original constitutional convention. There is very little opposition to the view that the National Guard must become more national, but even if this policy is pursued to a radical degree, it is by no means necessary to ignore the present Guard as a basis nor to do away with the system of organization by local units.

